# Parental experiences of stillbirth rituals in Limpopo, South Africa

**DOI:** 10.4102/hsag.v30i0.2961

**Published:** 2025-07-22

**Authors:** Lunghile Shivambo, Dumile Gumede

**Affiliations:** 1 Department of Development Studies, College of Human Sciences, University of South Africa, Pretoria, South Africa; 2 Department of Nursing, Faculty of Health Sciences, Durban University of Technology, Durban, South Africa

**Keywords:** stillbirth, burial practices, parents, cultural relativism, Limpopo, South Africa

## Abstract

**Background:**

Stillbirth remains a significant global health issue. Although extensive literature exists on burial practices for live births, there is a notable gap in evidence regarding the specific cultural practices related to the burial of stillborn infants.

**Aim:**

This study examines the burial practices surrounding stillbirth from the perspectives of parents using the lens of cultural relativism.

**Setting:**

The study was conducted in the Mopani District, South Africa.

**Methods:**

A qualitative approach was used to conduct in-depth interviews with 12 parents who had experienced stillbirth. Data were analysed using thematic analysis, focusing on burial rituals, beliefs and the meaning of these practices in helping parents process their grief. The Consolidated Criteria for Reporting Qualitative Research (COREQ) checklist was used in preparing the manuscript.

**Results:**

Participants described several burial practices performed following stillbirth including preparation of the body of a stillborn infant, selection of a burial site for a stillborn infant, the timing of burial for a stillborn infant, cultural prohibition on men’s attendance at the burial of a stillborn infant and sexual practices observed during the mourning period. The cultural significance of these practices was highlighted in terms of subsequent pregnancy.

**Conclusion:**

Burial practices performed for stillborn infants are essential for parents to recover from grieving and enable them to hope for a successful subsequent pregnancy. These practices should be respected and integrated into the broader healthcare framework to provide culturally competent bereavement care.

**Contribution:**

The study offers an interdisciplinary understanding of the intersection between culture and stillbirth.

## Introduction

Stillbirths – babies born with no sign of life at 28 weeks of pregnancy or later (World Health Organization [WHO] 2019) – are a global public health challenge, and the majority occur in lower- and middle-income countries (LMICs) (Mukherjee et al. 2024). Globally, in 2019, around two million babies were stillborn (Hug et al. 2021), of which 84% occur in LMICs (UN IGME 2020). In South Africa, approximately 16 000 stillbirths occur annually (Hlongwane et al. 2022), with persistently high rates reported in rural areas such as Limpopo province (Makhado 2018). Within these rural communities, cultural practices play a central role in guiding through burial processes following a stillbirth. These burial practices are deeply embedded in the social and cultural fabric of the community and offer an alternative form of support when medical or psychological resources may be limited. Stillbirth is one of the most challenging and traumatic experiences and has life-long impacts on parents (Nuzum, Meaney & O’Donoghue 2017), together with substantial psychological, social and financial consequences (Cacciatore 2013; Heazell et al. 2016; Pollock et al. 2020). However, despite this significant burden of stillbirths on the bereaved parents, cultural understandings surrounding stillbirths in LMICs are scarce.

Cultural values and beliefs are vital in performing burial practices for stillborn infants and attaching meanings associated with the practices (Arach et al. 2023). Burial practices surrounding stillbirth remain a deeply personal and culturally specific aspect of bereavement, reflecting broader societal beliefs about life, death and the status of stillborn infants (Ayebare et al. 2021). Burial practices vary across cultures. In many cultures, stillbirth is surrounded by silence and social stigma (Ayebare et al. 2021; Sun et al. 2020) often leaving parents without proper guidance or support during their grieving process. Globally, research has largely focused on the emotional and psychological impacts of stillbirth (Burden et al. 2016; Cacciatore 2013) with limited attention to the rituals and cultural practices that may shape parents’ grieving process and social experiences. This gap in understanding is particularly evident in rural South African contexts, where traditional and modern beliefs often intersect.

The prevailing customs of the parents’ cultural environment determine the burial practices performed for a stillborn infant. Generally, burial practices include burial methods, for example, in-ground burial and cremation, burial site selection (Arach et al. 2023), burial timing (Kiguli et al. 2015; Mills et al. 2023), body preparation (Sun et al. 2020), gender-specific roles in the burial process (Ayebare et al. 2021) and mourning period. For instance, in some cultures, stillborn babies are not considered fully human and are thus buried secretly (Ayebare et al. 2021; Haws et al. 2010), thus perpetuating the social stigma surrounding stillbirths. A study in Northern Uganda reported that stillborn babies were dressed in a white cloth as a sign of love and holiness (Arach et al. 2023). Another qualitative study of health workers’ experiences of caring for parents after stillbirth in Kenya and Uganda reported that rapid burial was a cultural norm in both countries (Mills et al. 2023). This suggests that burial practices are determined and shaped by cultural values and beliefs.

Although extensive literature exists on burial practices for live births, there is a notable gap in evidence regarding the specific cultural practices associated with the burial of stillborn infants. Yet, mourning rituals and burial practices are different for stillborn babies as compared to other deaths (Ayebare et al. 2021). This study is important because it sheds light on the intersection of culture, grief and gender dynamics, offering insights into how these burial practices shape the grieving process. This study aims to fill this gap by examining cultural practices surrounding stillbirth from the perspectives of men and women in South Africa and to provide a balanced perspective on how societal norms and individual experiences intertwine in the aftermath of stillbirth.

Cultural relativism is a sociological and anthropological theoretical approach that promotes understanding cultural practices within their own context (Geertz 1973). It offers an insightful framework for studying burial practices in non-Western societies that encourages understanding people’s behaviours, beliefs and practices in their cultural contexts (Geertz 1973). This perspective challenges the ethnocentric notion that grief and burial systems must adhere to Western norms and acknowledges the significance of cultural rituals in emotional healing, burial and grief management. In other words, cultural relativism emphasises that practices must be understood within their specific cultural contexts without imposing external judgements. Such an approach allows researchers to honour local customs and values while exploring their impact on grieving parents. Rather than viewing these practices through a lens of ‘right’ or ‘wrong’, cultural relativism seeks to understand their role in the recognition of cultural differences (Van der Merwe 1999).

Therefore, understanding cultural practices associated with the burial of stillborn infants is essential for informing culturally sensitive bereavement psychosocial support interventions and policies that are inclusive of diverse cultural perspectives. This study emphasises the need to document and analyse these practices to support culturally informed healthcare interventions, reduce stigma and improve emotional outcomes for bereaved families in South Africa. By recognising the cultural context of stillbirth-related practices, healthcare providers, researchers and policymakers can address the emotional, social and spiritual needs of grieving parents with greater sensitivity and effectiveness.

The purpose of this study was to examine the burial practices for stillborn infants from the perspectives of men and women (parents), and the meaning these practices hold within the context of Xitsonga culture in Limpopo, South Africa, using the lens of cultural relativism.

## Research design and methodology

### Study design

This study adopted a qualitative approach to understand the burial practices for stillborn infants. Qualitative research is a method of inquiry that seeks to understand human experiences, behaviours and interactions by exploring them in-depth (Merriam & Tisdell 2016). A descriptive phenomenological design (Creswell 2007) was considered appropriate for this study as it enables an in-depth exploration of the complex, culturally embedded practices, beliefs and emotional responses surrounding stillbirth. This approach also allows for a nuanced understanding of how cultural practices influence participants’ experiences of grief, coping mechanisms and support needs.

### Setting

The selected study area was Muyexe village in the Mopani district, Limpopo province, South Africa. The area is situated closer to the Kruger National Park in Shingwedzi Camp. The village is highly populated with Xitsonga ethnic individuals. A total of 392 stillbirths occurred from 01 February 2018 to 31 October 2021 at Kgapane Hospital in Mopani district (Marincowitz & Marincowitz 2024), reflecting that stillbirths are a public health challenge in the province.

### Participants and sampling

The study population included 12 participants (8 women and 4 men) who were aged 18 years and above and had experienced a stillbirth. The inclusion criteria were women who had at least one stillbirth and men whose partners had at least one stillbirth, residing in the study area, and willing to participate in the study. Those who did not meet the inclusion criteria were excluded from the study. Participants were selected through purposive and snowball sampling techniques (Merriam & Tisdell 2016) to ensure that they had experienced the event and were willing to discuss in a culturally sensitive manner the burial practices that were performed for their stillborn infants. Purposive sampling was initially used to select three participants, after which the snowball sampling was employed to recruit additional participants through referrals from those already selected. Data collection continued until saturation was reached after 12 interviews, as no new themes or insights emerged, indicating that the phenomenon was sufficiently explored (Patton 2015).

### Data collection

Participants were recruited in the community with the assistance of a home-based care worker from the local primary healthcare facility. Home-based care workers were deemed strategically positioned as they visited households for community health services and were regarded as trusted community members.

Data were collected through face-to-face, in-depth interviews conducted in the participants’ home language (Xitsonga) to capture in-depth narratives about burial practices surrounding stillbirth within the Xitsonga culture and the meanings these parents ascribe to them. Demographic data, including age, marital and employment status, education level and number of stillbirths and children, were collected to contextualise participants’ experiences. In qualitative health research, in-depth interviews study the experiences of participants and the meaning of diseases and health outcomes they attach to them (Tong, Sainsbury & Craig 2007). The interviews were supplemented by field notes taken during the interviews. In-depth interviews were chosen to encourage participants to share their own experiences and meanings without the influence of others (Merriam & Tisdell 2016). An interview guide was used to conduct the interviews, and it consisted of open-ended questions to guide the interviews and focused on:

- The burial practices performed following a stillbirth for a stillborn infant.
- The meaning of these practices.
- The influence of these practices on their grieving process.

The first author, LS, conducted in-depth interviews and was a member of the Xitsonga culture. The first author had witnessed some beliefs and practices related to stillbirth in the study area. With this background, the first author employed reflexivity (Berger 2015) to avoid personal bias and to set aside their preconceived knowledge about the burial practices and meanings associated with these practices. The second co-author, DG, a social scientist, was an ‘outsider’ to the culture, which helped in debriefing and identifying the gaps that were filled during the data analysis and report writing (Arach et al. 2023).

Data collection was conducted at participants’ homes between August and September 2023. Interviews were audio-recorded to ensure accurate data capture and lasted between 30 min and 60 min. Interviews were conducted until saturation was reached, which occurred after 12 interviews, as there was no new information arising on the different sub-topic areas from the addition of participants (Arach et al. 2023; Guest, Bunce & Johnson 2006).

### Data analysis

Before analysis, LS listened to the audio recordings more than once and transcribed them verbatim in Xitsonga. LS also translated the Xitsonga transcripts into English. Data collection and analysis were conducted concurrently to allow emerging insights to inform subsequent interviews, thereby enhancing the depth and relevance of the data collected. LS cross-checked each transcript with the audio recording to ensure the accuracy, consistency and completeness of the data collected (Arach et al. 2023). DG supervised both the data collection and analysis processes to ensure methodological rigour and consistency. The English transcripts were then saved on a computer and later uploaded into ATLAS.ti Version 23 for coding and generation of themes.

Data were analysed using thematic analysis by following a 10-step approach for conducting qualitative data analysis in health sciences (Babchuk 2019). The 10-step approach includes:

(1) Assembling materials for analysis, (2) refamiliarising oneself with the data, (3) open or initial coding procedures, (4) generating categories and assigning codes to them, (5) generating themes from categories, (6) strategies of validation, (7) interpreting and reporting findings from the participants, (8) interpreting and reporting findings from the literature, (9) visual representations of data and findings, and (10) strengths, limitations and suggestions for future research. (Babchuk 2019)

Both inductive and deductive analysis approaches were employed. The analysis was guided by the principles of cultural relativism (Geertz 1973) ensuring that burial practices were understood within the context of the community and not evaluated through external cultural norms.

The whole process of analysis was carried out by the first author, LS, and then later verified by the second co-author, DG. Participants’ quotes were used to support the results. In addition, the study adhered to the Consolidated Criteria for Reporting Qualitative Research (COREQ) guidelines, ensuring methodological rigour and transparency (Tong et al. 2007).

### Trustworthiness of the study

The trustworthiness of this study was ensured by adhering to the criteria of credibility, transferability, dependability and confirmability (Lincoln & Guba 1985). Credibility was enhanced through the triangulation of data sources (in-depth interviews and field notes) and peer debriefing to validate findings and minimise researcher bias. Transferability was supported by providing rich, contextual descriptions to allow readers to assess the applicability of the findings to other settings. Dependability was maintained through a clear audit trail documenting the research process, while confirmability was strengthened by practising reflexivity and ensuring that findings were grounded in the participants’ accounts rather than researcher assumptions.

### Ethical considerations

An application for full ethical approval was made to the Research Ethics Committee at a public South African university, and ethics consent was received (reference no: 64079228_CREC_CHS_2023). Gatekeeper permission to conduct the study in the community was obtained from the traditional council. Written informed consent was obtained from all participants before the start of each interview and for audio recording and publishing of study findings, to which all participants agreed. Each participant was assigned a unique anonymous identifier to enhance confidentiality. In addition, participants were provided with a referral pathway in case distressed participants needed professional counselling. Of the 12 participants, only 2 were referred to a social worker. The remaining 10 reported no need for professional counselling. All procedures performed in studies involving human participants followed the ethical standards of the institutional research committee and the 1964 Helsinki Declaration and its later amendments or comparable ethical standards.

## Results

### Characteristics of the study participants

The study included 12 participants who had experienced stillbirths, with diverse demographic and socio-economic profiles. Participants ranged in age from 23 years to 61 years, with most in their late 20s to 50s. The group included eight women (F) and four men (M), capturing both maternal and paternal perspectives on stillbirth. Seven participants were married, and five were unmarried, including couples who lost the same child, offering insights from both partnered and single parents. Employment varied: four were employed full-time, three held part-time or contract roles, four were unemployed and one was self-employed. Educational levels ranged from Grade 2 to Grade 12, with most having completed secondary education. Participants had experienced one to two stillbirths and reported between zero and six live births, reflecting diverse reproductive histories and family dynamics. The sample included individuals from varied socio-economic and educational backgrounds, capturing a broad spectrum of lived experiences related to stillbirth in rural South Africa. This diversity enriches the study’s understanding of how demographic and contextual factors influence burial practices following stillbirth.

### Burial practices for stillborn infants

Participants described five key burial practices that were performed following stillbirth, each representing distinct yet interconnected cultural responses to stillbirth. These include: (1) preparation of the body of a stillborn infant, (2) selection of a burial site for a stillborn infant, (3) the timing of burial for a stillborn infant, (4) cultural prohibition on men’s attendance at the burial of a stillborn infant and (5) sexual practices observed during the mourning period. Together, these burial practices illustrate the profound role of culture in shaping the grieving process and facilitating emotional healing for parents and their communities.

### Preparation of the body of a stillborn infant

Participants described various practices involved in preparing the body of a stillborn infant for burial. These practices varied; for example, some involved bathing or dressing the body, while others did not. The clothing choices were often carefully selected, representing the parents’ hopes, love or personal significance:

‘No, they don’t bathe a small baby, but when it comes to dressing, I believe it’s up to the family. If you’ve bought clothes for the baby, you can dress them, but with ours, we didn’t. We just wrapped him in a blanket at the hospital. What I do know is that the baby shouldn’t be buried naked; they should either be wrapped or covered with something.’ (P06, F, 47 years old)

‘I believe dressing the baby is based on the mother’s preference, but no, they don’t bathe the baby. As a mother who was planning to have a baby, you would naturally buy some clothes and pack them at home in preparation for the birth. Unfortunately, in my case, I had to choose one of the best outfits I had bought before the baby was born and give it to my aunts so they could dress the baby.’ (P02, F, 38 years old)

‘They then brought the baby to me so I could see and properly wrap him, after which they placed him in the coffin. However, I did not attend the burial as I was still in pain, so I am unsure of how the baby was laid to rest.’ (P03, F, 28 years old)

In contrast, the practice of dressing a stillborn infant was not observed by all participants. One woman said:

‘They said that we could not bathe or dress a small baby because, if we did, I would never be able to conceive again.’ (P04, F, 39 years old)

The results indicate that in this cultural context, there was a belief tied to the notion that bathing and dressing a stillborn infant could have a significant impact on the mother’s future ability to conceive, as P04 was advised that bathing or dressing the baby may prevent future pregnancies. This highlights the meaning attached to the practice of neither bathing nor dressing the body of a stillborn infant for burial.

### Selection of a burial gravesite for a stillborn infant

The selection of a burial gravesite for a stillborn infant was a deeply personal and emotional process influenced by cultural norms. Participants stated that, following Xitsonga culture, a burial gravesite for a stillborn infant is either within the homestead yard or next to a stream or river where water is flowing. One woman said:

‘No, babies are not buried in the cemetery; they are buried next to a stream. He was laid to rest beside a flowing stream.’ (P05, F, 51 years old)

A man stated:

‘They brought the baby home and buried her in the yard, right behind the house.’ (P12, M, 54 years old)

Participants’ accounts highlighted the influential role of cultural beliefs in shaping the choice of burial sites for stillborn infants.

### The timing of burial for a stillborn infant

The timing of burial for a stillborn infant refers to when the burial or other funeral arrangements for a stillborn infant take place following the stillbirth. Participants reported that the timing of burial for stillborn infants is determined according to Xitsonga culture. Thus, they explained that their stillborn infants were buried on the same day they were born dead. One woman explained:

‘On the same day, since the baby passed away in the early hours, all the arrangements were made throughout the morning. He was buried that very day, as I was also discharged from the hospital that day because I wasn’t bleeding excessively.’ (P05, F, 51 years old)

‘It doesn’t take long to bury a baby. Both of my babies were laid to rest on the same day they passed away.’ (P01, F, 57 years old)

Participants’ accounts highlighted the cultural norm within Xitsonga tradition of burying stillborn infants on the same day of their birth, reflecting both practical considerations and deeply held beliefs about mourning and closure.

### Cultural prohibition on the men’s attendance at the burial of a stillborn infant

Several participants mentioned that fathers of stillborn infants are prohibited from attending a burial for their stillborn infants. It was stated that the prohibition of a man who is the father of a stillborn infant was a belief that non-attendance at the burial would enable subsequent pregnancy for the mother of a stillborn infant. The differences in how men and women experienced the burial process were shaped by cultural expectations and societal norms, as illustrated by three men:

‘I’m not sure what happens there, as men are not allowed, but the day after the baby is buried, they take the father or other male family members to see the grave … Before the burial, I arranged a tent and chairs.’ (P07, M, 61 years old)

‘No, I was not allowed. They told me I couldn’t be there during the burial. The only parent permitted on that side is the mother.’ (P09, M, 38 years old)

‘The burial process was managed by the grandmothers. Once everything was arranged at the hospital, the funeral preparations began there. When the body was brought home, the elderly women took charge and oversaw the entire process. Not everyone attended; it was just a small group – my girlfriend, her mother, my mother, a few female relatives, and the elderly women.’ (P11, M, 24 years old)

In addition, participants described various cultural rituals performed by women during the burial of a stillborn infant, such as wrapping the body in a blanket instead of using a coffin and filling the grave using their waist rather than tools. Elderly women played a key role in these practices, which were believed to promote future successful pregnancies. Three women shared their experiences as follows:

‘Once we reached the graveside, the elderly women gave me instructions. They told me to pee on the grave of the baby to ensure I would be able to have more children if I wanted to. I followed their advice, and here I am today, having had three more children since then. From my perspective, everything was done according to tradition, and it worked – I’ve had more children, and they’re all healthy.’ (P01, F, 57 years old)

‘Once again, it was the elderly women who dug the grave. As the mother of the deceased baby, I was instructed to urinate in the grave and then use my waist to push the soil back into the grave, as a ritual to ensure I would be able to have children in the future.’ (P04, F, 39 years old)

‘When we arrived at the gravesite, they broke the small coffin into pieces, removed the baby, wrapped him carefully, and placed him in the grave without the coffin. They then placed the pieces of the coffin on top of the baby. I’m not sure if this is the way it’s done for everyone who loses a baby, but that was the process followed during my baby’s burial.’ (P08, F, 32 years old)

These practices were followed in the cases described and were seen as an essential part of the burial process, reflecting cultural beliefs and traditions surrounding fertility and closure.

### Sexual practices observed during the mourning period

Participants explained the sexual practices among bereaved parents of a stillborn infant, including sexual abstinence during the mourning period. Cultural norms prescribed a period of abstinence before the bereaved parents could engage in sexual activities. One woman said:

‘They told me I should not be intimate with any man during those three months of mourning.’ (P03, F, 28 years old)

One man stated:

‘Yes, we are Black, and there are certain cultural practices we must follow. After the funeral, the elders called us and informed us that we had to refrain from being intimate for six months. They also tied a small rope around her waist for that period. Once the six months were up, they would remove the rope and allow us to resume our life as husband and wife.’ (P07, M, 61 years old)

Another said:

‘The elderly women from our families and church called my girlfriend and me together and told us that we needed to mourn for three months. They advised us to give ourselves time to grieve and process everything and also for her body to cleanse during that time, meaning she should have her period three times before we could be intimate. They also emphasized the importance of her coming to terms with the loss of the baby.’ (P11, M, 24 years old)

It was clear from the participants that sexual abstinence was intended to allow both emotional and physical healing and to help the mother come to terms with the loss of the baby before resuming normal marital life.

## Discussion

This study provides a detailed examination of the cultural practices surrounding the preparation and burial of stillborn infants within a Xitsonga community. Using a cultural relativism approach (Geertz 1973), the study explores these practices within the context of deeply ingrained cultural beliefs, highlighting how they offer meaning and closure to the bereaved parents. Understanding these practices requires viewing them from within the culture, recognising that they may not align with global or medical norms but that they hold significant emotional and social meaning in their own right.

The findings of this study highlight the meaningful role of the family, particularly in dressing the stillborn infant in a chosen outfit for burial. This practice was filled with emotional significance, symbolising the family’s hopes and love for the child that was not to be. This aligns with other studies that highlight the emotional weight of rituals surrounding the preparation of a deceased infant, where the handling of the body becomes a key aspect of mourning (Black 2020; Mowll et al. 2022; Ryninks et al. 2014). However, there were cases where mothers chose not to dress their infants, opting instead for wrapping them in a blanket, further illustrating the variance within the practice that is influenced by personal preference and emotional state.

Interestingly, the belief that a stillborn infant should not be buried naked was consistent across participants, suggesting a cultural imperative to cover the body. This aligns with findings in other cultures where the body of the deceased is treated with utmost respect, as it is believed that the soul’s journey to the afterlife is dependent on these rites (Joarder, Cooper & Zaman 2014). However, the practice of dressing up a stillborn infant for burial is not universal. A study in Taiwan reported that out of the 20 stillborn babies, 19 (95%) were naked, and only 1 (5%) was covered with a piece of cloth for burial (Sun et al. 2020). In this study, a participant also described a prohibition on bathing or dressing the stillborn baby because of a belief that doing so could hinder future fertility, illustrating the complex intersection between cultural practices and personal or familial fertility beliefs. This cultural restriction is indicative of practices in other societies where taboos around the handling of stillborn are tied to beliefs about the health and fertility of the mother (Ayebare et al. 2021).

The selection of a burial site for stillborn infants was another culturally significant practice explored in the study. Participants revealed that, in line with Xitsonga traditions, stillborn infants are often buried in locations outside conventional cemeteries, such as next to a stream or within the homestead yard. This practice of burial within the homestead yard is influenced by the belief in maintaining a close connection with the deceased and the desire for the child to rest in a peaceful, natural environment (Arach et al. 2023). Studies in similar cultural contexts suggest that burial sites are chosen not only for practical reasons but also for their symbolic value, reinforcing the emotional bond between the family and the deceased (Arach et al. 2023; Ayebare et al. 2021). Literature shows that, in some cultures, the selection of the burial gravesite relied on the culture and marital status of the parents (Ayebare et al. 2021). For instance, a qualitative study done in Northern Uganda reported that burial sites were located at the paternal homes for stillborn infants (Arach et al. 2023).

The timing of the burial is dictated by both cultural expectations and practical considerations, with stillborn infants being buried on the same day as their delivery in most cases. This practice speaks to the urgency of processing the loss and providing the infant with a dignified final resting place. The findings corroborate those of other studies, which have noted the importance of quick burial in many African cultures, often linked to beliefs about the spirit’s journey (Bedwell et al. 2023; Kiguli et al. 2015; Mills et al. 2023). The rapid arrangements following the death highlight the significance of timely closure for the family, particularly the mother, who may experience a significant emotional toll. The fact that one mother was discharged from the hospital the same day as the infant’s delivery, indicates a desire to expedite the grieving process; reflecting cultural norms around immediate mourning and burial practices. While cultural practices often require the urgent burial of stillborn babies, this urgency can hinder mothers from achieving closure, especially when physical pain or medical recovery prevents them from attending. This highlights how well-intentioned traditions may unintentionally impede emotional healing for bereaved mothers.

A striking cultural practice uncovered in this study is the prohibition of fathers from attending the burial of a stillborn infant. The exclusion of men from the burial ritual speaks to broader gender dynamics and expectations within the community. This practice appears to be rooted in beliefs about the impact of the father’s presence on the mother’s future fertility (Ayebare et al. 2021). Fathers are often excluded from certain mourning rituals, with the underlying belief being that their presence might affect the mother’s ability to conceive again, thus making the exclusion an act to preserve the possibility of future pregnancies.

The study’s findings revealed that gender-specific roles are prominently observed in the burial of a stillborn infant within Xitsonga culture. Fathers were primarily responsible for making funeral arrangements, such as organising burial logistics and obtaining necessary permissions. The focus on women as primary mourners and the role of elderly women in overseeing the burial also aligns with the idea of preserving cultural continuity through female-centred rituals. Elderly women are seen as custodians of cultural knowledge, managing the burial ritual process and ensuring its adherence to tradition (Arach et al. 2023). The findings corroborate those of other studies, which have noted the role of older women in the burial of stillborn infants (Ayebare et al. 2021; Kiguli et al. 2015). The involvement of older women in leading the burial processes for stillborn infants is informed by a belief that those who have passed the childbearing age would enable future fertility and avoid the potential for the recurrence of pregnancy loss (Ayebare et al. 2021). These roles highlight the societal norms dictating gendered responsibilities in performing burial practices associated with stillbirth, revealing distinct approaches to addressing grief within family and community contexts.

The findings of the study indicate that, as part of the grieving and healing process, parents were expected to observe sexual abstinence during the mourning period following the stillbirth. In contrast, in another study in which there were no cultural norms around the sexual activities of bereaved parents, participants reported that their sexual relations were reduced during the mourning period as there was a fear of subsequent pregnancy and having to go through another stillbirth (Camacho-Avila et al. 2023). However, in this study, this ritualised approach to mourning through sexual abstinence is not only about grieving but also about re-establishing balance and fertility in the family, an act of restoring harmony in the aftermath of a loss.

### Recommendations

The importance of rituals such as sexual abstinence, mourning periods and symbolic acts should be respected, but it is equally important that these practices are not seen as barriers to the well-being of the mother. Healthcare providers should ensure that mothers’ emotional and physical needs are met during and after the mourning period, offering guidance and support in balancing cultural practices with personal health. Moreover, it is important for community leaders, including traditional elders and religious figures, to engage in discussions around stillbirth mourning and burial practices, particularly to address any potentially harmful or limiting practices.

### Limitations

The study is based on a limited sample of participants, primarily drawn from a specific Xitsonga cultural context. While the study offers rich qualitative data, the small sample size and the lack of geographic or cultural diversity limit the generalisability of the findings. Future research could include a larger and more diverse sample to capture variations within and across different African cultural groups. In addition, while the study touches on the emotional significance of the burial rituals, there is limited exploration of the long-term emotional impact on bereaved parents. Further studies could focus on how the practices help or hinder emotional healing over time, particularly for women who experience both grief and physical recovery after stillbirth.

### Areas for future studies

This study found that there are clear gender differences in the mourning and burial process, with women assuming primary responsibility for rituals and men being excluded from the burial. Future research could explore the broader implications of these gendered practices on emotional healing, marital relationships and family dynamics. Understanding how these cultural norms affect the emotional well-being of fathers and their relationships with the mothers of their children could help address the emotional needs of men during the mourning period. In addition, given that cultural practices related to fertility and mourning were frequently mentioned, future studies could explore the long-term impact of these practices on women’s fertility and emotional health. Specifically, understanding whether these cultural beliefs have a positive or negative impact on women’s overall well-being could provide valuable insight for healthcare providers.

## Conclusion

This study has highlighted the complex interplay of cultural beliefs, emotional needs and societal norms that shape the mourning and burial practices for stillborn infants in the Xitsonga community. Understanding these practices through the lens of cultural relativism, provides insights into the importance of ritual in providing closure, promoting emotional healing and preserving fertility. While these practices may appear unconventional from a Western perspective, they serve essential roles within their cultural context, ensuring that the family, particularly the parents, can process grief, restore emotional balance and continue with life. The study’s recommendations aim to promote a more culturally sensitive and supportive environment for parents dealing with the loss of a stillborn infant, ensuring that both traditional and medical care are integrated. Healthcare systems should recognise and respect these practices, integrating them into bereavement care to ensure culturally sensitive support for grieving parents. Future research into the long-term effects of these cultural practices on fertility, emotional healing and gender dynamics will help enrich our understanding of the intersections between cultural beliefs and reproductive health.
